# Spontaneous cognition in dysphoria: reduced positive bias in imagining the future

**DOI:** 10.1007/s00426-018-1071-y

**Published:** 2018-08-10

**Authors:** Julie L. Ji, Emily A. Holmes, Colin MacLeod, Fionnuala C. Murphy

**Affiliations:** 1 Centre for the Advancement of Research on Emotion, School of Psychological Science (M304), University of Western Australia, 35 Stirling Hwy, Crawley, 6009 WA Australia; 2 Department for Clinical Neuroscience, Karolinska Institutet, Berzelius väg 3, Solna, Stockholm, Sweden; 3 Medical Research Council Cognition and Brain Sciences Unit, University of Cambridge, 15 Chaucer Rd, Cambridge, UK

**Keywords:** Dysphoria, Spontaneous future thinking, Mental time travel, Mindwandering, Mental imagery, Cognitive bias

## Abstract

Anomalies in future-oriented cognition are implicated in the maintenance of emotional disturbance within cognitive models of depression. Thinking about the future can involve mental imagery or verbal-linguistic mental representations. Research suggests that future thinking involving imagery representations may disproportionately impact on-going emotional experience in daily life relative to future thinking not involving imagery (verbal-linguistic representation only). However, while higher depression symptoms (dysphoria) are associated with impaired ability to deliberately generate positive relatively to negative imagery representations of the future (when instructed to do so), it is unclear whether dysphoria is associated with impairments in the tendency to do so spontaneously (when not instructed to deliberately generate task unrelated cognition of any kind). The present study investigated dysphoria-linked individual differences in the tendency to experience spontaneous future-oriented cognition as a function of emotional valence and representational format. Individuals varying in dysphoria level reported the occurrence of task unrelated thoughts (TUTs) in real time while completing a sustained attention go/no-go task, during exposure to auditory cues. Results indicate higher levels of dysphoria were associated with lower levels of positive bias in the number of imagery-based future TUTs reported, reflecting higher negative imagery-based future TUT generation (medium to large effect size), and lower positive imagery-based TUT generation (small to medium effect size). Further, this dysphoria-linked bias appeared to be specific in temporal orientation (future, not past) and representational format (imagery, not non-imagery). Reduced tendency to engage in positive relative to negative imagery-based future thinking appears to be implicated in dysphoria.

**Electronic supplementary material:**

The online version of this article (10.1007/s00426-018-1071-y) contains supplementary material, which is available to authorized users.

## Introduction

Disproportionately negative future-oriented cognition is implicated in the aetiology and maintenance of emotional disturbance within cognitive models of depression (Abramson, Metalsky, & Alloy, 1989; Beck, 1976; Beck, Brown, Steer, Eidelson, & Riskind, 1987). Empirical evidence has shown that, when asked to deliberately evaluate or anticipate future events, individuals with depression exhibit pessimistic judgments of their personal futures (Alloy & Ahrens, 1987; MacLeod et al., 2005; Miranda & Mennin, 2007), and show impaired ability to anticipate positive relative to negative future events (Holmes, Lang, Moulds, & Steele, 2008; MacLeod & Conway, 2007; MacLeod & Salaminiou, 2001; Morina, Deeprose, Pusowski, Schmid, & Holmes, 2011). However, research suggests that the emotional consequences of mental representations of future events are greater when such representations occur spontaneously, without instruction or intention, relative to when such representations were generated deliberately (Cole, Staugaard, & Berntsen, 2016). Further, experience sampling research suggests that the emotional consequences of future-oriented mental representations appear to be restricted to those involving mental imagery (Barsics, Van der Linden, & D’Argembeau, 2016). As such, understanding biases in the tendency to spontaneously engage in mental imagery-based emotional future thinking may further illuminate the link between future-oriented cognition and emotional disturbance in depression.

### Mental imagery can evoke emotional response in an as-if-real manner

Consciously experienced mental representations can occur in mental imagery format, or verbal-linguistic format (Paivio, 1990). Mental imagery refers to internal representations of sensory-perceptual information in the absence of external sensory input, often referred to as “‘seeing from the mind’s eye’, ‘hearing from the mind’s ear’, and so on” (Kosslyn, Ganis, & Thompson, 2001, p. 635). Due to the recruitment of common neural substrates during mental imagery and sensory perception (Cichy, Heinzle, & Haynes, 2012; Klein et al., 2004; Kosslyn, 1994; Pearson & Kosslyn, 2015), mental imagery has the capacity to simulate not only the perceptual details of hypothetical events, but also the experiential correlates of such experiences in an as-if-real manner (Lang, 1979; Moulton & Kosslyn, 2011). Research has demonstrated the capacity for imagery representations of emotion stimuli to evoke emotional response at subjective, physiological, and neural levels (see Ji, Heyes, MacLeod, & Holmes, 2016). In healthy individuals, many studies have demonstrated that when asked to deliberately engage in mental imagery representations relative to verbal-linguistic representations of negative and positive information, imagery representations tend to evoke greater emotional response, at least at subjective levels (Holmes, Lang, & Shah, 2009; Holmes & Mathews, 2005; Holmes, Mathews, Mackintosh, & Dalgleish, 2008; Mathews, Ridgeway, & Holmes, 2013; Nelis, Vanbrabant, Holmes, & Raes, 2012).

Maladaptive cognition implicated in emotional disorders often involves mental imagery, although the focus has been on anxiety related disorders (Holmes & Mathews, 2010). Seminal theories of depression refer to both “thoughts and visual images” (p. 8) as relevant factors in the aetiology and treatment of depression (Beck, Rush, Shaw, & Emergy, 1979). More recently, a large questionnaire-based study investigating the properties of depressive thoughts found that more than half of 403 depressed patients reported their depressive thoughts to involve auditory and visual mental imagery (Moritz et al., 2014), suggesting that cognition involving mental imagery may be a prevalent feature of maladaptive cognition in depression too. Given the capacity for mental imagery to strongly impact emotional experience, investigating depression-linked biases in the tendency to experience mental representations involving mental imagery (whether consisting of imagery only, or a combination of imagery and verbal thought) is of particular importance for understanding the cognitive basis of such emotional psychopathology.

### Spontaneous future imagery-evoked emotional response

More recently, experience sampling research assessing the real-time occurrence of future-oriented thoughts and emotional states throughout the day has shown that individuals are more likely to experience negative or positive thoughts about the future following experiences of negative or positive emotional states, respectively (Barsics et al., 2016). In turn, the occurrence of such negative or positive thoughts about the future are associated with increases in subsequent experiences of negative and positive emotional states, indicating a bi-directional relationship between future-oriented cognition and state emotion (Barsics et al., 2016). An important aspect of Barsics et al.’s finding is that this relationship was restricted to emotional future-oriented cognition involving mental imagery, as the occurrence of emotional future cognition not involving mental imagery (i.e. verbal-linguistic thought) was not related to subsequent emotional states. As such, higher levels of negative, or indeed depressed, mood may arise, and be maintained by, a disproportionate tendency to spontaneously engage in negative relative to positive future thinking, particularly if such thinking involves mental imagery.

### Biases in mental imagery-based future thinking in depression

Abnormalities in mental imagery-based cognition have been implicated in depression, indicating elevated accessibility of imagery-based mental representations and reduced accessibility of positive imagery-based mental representations (Holmes, Blackwell, Burnett Heyes, Renner, & Raes, 2016). For example, when asked to deliberately anticipate negative and positive events in one’s personal future, individuals with higher depression symptoms are able to anticipate more negative future experiences and fewer positive future experiences than those with lower depression symptoms (MacLeod & Byrne, 1996; MacLeod & Salaminiou, 2001; for a review see MacLeod, 2016). When asked to deliberately generate imagery representations of future events in response to cues, higher depression symptoms are associated with reduced specificity of future events, particularly in response to positive cues (Hallford, Austin, Takano, & Raes, 2018), and reduced imagery vividness for positive relative to negative events (Holmes, et al., 2008; Morina et al., 2011; Szőllősi, Pajkossy, & Racsmány, 2015).

Reduced ability to deliberately generate imagery-based positive relative to negative representations of the future could potentially explain why individuals with higher depression symptoms have a reduced positive mood and elevated negative mood. However, depression-linked individual differences in the relative ability to generate positive and negative future imageries in response to direct instruction may not necessarily align with depression-linked individual differences in the relative tendency to do so without such instructions. Laboratory research in healthy individuals has shown that the emotional impact of spontaneously generated mental imagery representations of future events is greater relative to deliberately generated mental imagery representations of future events (Cole et al., 2016). Therefore, investigating depression-linked biases in the tendency to engage in emotional future imagery may further illuminate the role of future thinking and emotional disturbance in depression.

### Limited understanding of spontaneous mental imagery-based future thinking in depression

Compared to research on the ability to generate future imagery in depression when deliberately instructed to generate such imagery (deliberate generation), research on depression-linked tendency to generate future imagery when not instructed to do so (spontaneous generation) is relatively neglected. In community samples, higher levels of depression symptoms appear to be related to higher frequency of self-reported daily experiences of negative mental imagery, and less frequent experiences of positive imagery (Weßlau, Cloos, Höfling, & Steil, 2015). Patients with depression report frequently experiencing unwanted mental imagery in the form of spontaneously occurring emotionally distressing mental imagery of past and future experiences (Brewin, Gregory, Lipton, & Burgess, 2010; Moritz et al., 2014; Patel et al., 2007). However, these and other studies have tended to rely on retrospective estimations of the frequency of distressing mental imagery (Newby & Moulds, 2011; Patel et al., 2007; Reynolds & Brewin, 1998). Given that naturally occurring thoughts tend to be rapidly forgotten if their occurrence is not recorded immediately (Berntsen, 1996), such as in a thought diary or via a button press during a computer task, interpretation of retrospective reports of thought frequency is thus constrained. In contrast, assessing the frequency of cognition as it occurs in real time has the potential to provide a more accurate assessment of naturally occurring thought frequency.

Few laboratory studies have assessed depression-linked individual differences in spontaneous mental imagery-based future thinking frequency in real time. Previous research has shown that spontaneous thoughts tended to occur during states of diffused attention when the individual felt bored, tired, or unengaged with the task at hand (Berntsen, 1996; Kosslyn, Seger, Pani, Hillger, and Stephen, 1990; McVay & Kane, 2010). Therefore, laboratory studies designed to induce states of diffused attention are suitable for measuring task unrelated thought frequency, such as low-demand sustained attention to response tasks involving go/no-go tasks (Robertson, Manly, Andrade, Baddeley, & Yiend, 1997). In addition, previous research has shown that spontaneous cognition is often triggered by external cues (Kvavilashvili & Mandler, 2004; Plimpton, Patel, & Kvavilashvili, 2015). Therefore, exposing participants to standardised environmental cues is more appropriate for investigating depression-linked individual differences in spontaneous cognition frequency than experience sampling studies ‘in-the-wild’, particularly as depression symptoms are likely to influence the selection of environmental context in daily life.

Kvavilashvili and colleagues (Kvavilashvili & Schlagman, 2011; Schlagman & Kvavilashvili, 2008) developed a variant of the sustained attentional vigilance tasks that additionally exposed participants to visually presented verbal cues. Using this approach, studies have found no significant relationship between depression symptom level and the general frequency of future-oriented task unrelated thoughts (TUTs) (Hoffmann, Banzhaf, Kanske, Bermpohl, & Singer, 2016; Plimpton et al., 2015), although Hoffmann et al. (2016) found future-oriented TUTs to be more emotionally negative in depressed relative to non-depressed individuals. However, such studies did not examine the frequency of future-oriented TUTs as a function of their emotional valence. It is therefore unclear whether dysphoria level is related to the tendency to spontaneously generate more negative relative to positive future TUTs.

Most importantly, no study investigating depression-linked individual differences in spontaneous future thinking has addressed whether (and to what extent) these vary with representational format, i.e. emotional future representations that involve mental imagery versus those that do not. In addition, imagining hypothetical future experiences (episodic simulation) recruits the core neural networks supporting episodic memory of past experiences, involving the flexible recombination of relevant contextual and sensory features from past experiences (Addis, Wong, & Schacter, 2007; Schacter, Addis, & Buckner, 2007, 2008). Therefore, it would be important to understand whether any relationship observed between depression symptom level and the tendency to experience mental imagery-based emotional future TUTs are unique to future-oriented TUTs by examining the relationship between depression symptom level and the tendency to experience mental imagery-based emotional past TUTs. If the same pattern of relationship was found between depression symptom level and both past- and future-oriented TUTs, then effects are likely to reflect depression-linked biases in the tendency to engage in emotional mental time travel in general, rather than a bias specific to the tendency to imagine emotional futures.

### The present study

The present laboratory study aims to investigate individual differences in the tendency to engage in spontaneous mental imagery-based emotional future thinking in participants varying in the level of self-reported symptoms of dysphoria (as indicated by depression questionnaire scores).

Examining depression-linked individual differences in thought frequency requires a task that is capable of evoking sufficient variation in the occurrence of TUTs. While most previous studies have used intermittent and infrequent random thought probes to assess TUT occurrence, the limitation with the probe-caught sampling approach is the fixed and low number of data points obtainable during brief laboratory tasks (typically 15–20 min). This “probe-caught” method is able to capture only a fraction of actual task unrelated thoughts, and such an approach may not yield sufficient variability in valid data points for examining the presence of systematic relationships between an individuals’ emotional states and the occurrence of particular types of cognition (e.g. future-oriented emotional task unrelated thoughts involving mental imagery). In contrast, self-reporting occurrences of mindwandering have the potential capacity to capture a greater proportion of the occurrences of task unrelated cognition, increasing the chance of obtaining sufficient data points for group-level frequency comparisons of cognition by valence and mode using means or rank order analysis. As such, following previous research (Cole et al., 2016), the present study employed a “self-caught” method, where participants self-reported TUTs as they occurred.

A novel task unrelated thinking (TUT) task was developed based on the Sustained Attention to Response Task (SART) by Robertson, Manly, Andrade, Baddeley, & Yiend (1997). The standard SART is a go/no-go task, which involves the withholding of key presses to rare targets (one in nine trials being a no-go trial). The SART measures lapses in sustained attention as a result of shifts from controlled to automatic processing (Robertson et al., 1997), and is widely used in mindwandering research (Christoff, Gordon, Smallwood, Smith, & Schooler, 2009; Smallwood, Beach, & Schooler, 2008). Previous research has shown that the same task with a high target frequency (one in two) induces less mindwandering relative to the standard low target frequency version (one in nine) in both low and high dysphoria samples (Murphy, Macpherson, Manly, & Dunn, 2013). The present study therefore utilised the standard low target frequency SART to encourage mindwandering and the emergence of task unrelated thoughts.

Further, the SART was modified to include exposure to standardised external cues in the form of emotional and non-emotional words presented during the SART task. This novel variant of the SART is similar to another vigilance task involving the presentation of verbal cues, developed by Schlagman & Kvavilashvili (2008) and used in studies such as Plimpton et al. (2015). However, the present TUT task differs to that of Schlagman & Kvavilashvili (2008)’s cued TUT approach in two key aspects. First, while Schlagman & Kvavilashvili (2008) visually presented verbal cues in the centre of the screen on every trial and participants were instructed to ignore the cues as they were task irrelevant, this invariably introduced visual attentional competition with the main task (discriminating the identity of visually presented line patterns), thereby making the task more difficult and less monotonous. In addition, participants were asked to indicate during the task whether each TUT reported was related to a visually presented verbal cue, which invariably reduces the task irrelevance of cues and increases awareness of cues during the task. The present task therefore used aurally presented verbal cues and participants were not required to indicate whether their TUTs were related to cues. Participants reported the occurrence of self-caught TUTs in real time during a number digit vigilance task while simultaneously exposed to task contexts that were emotional (negative and positive verbal cues) and unemotional (neutral verbal cues). To rule out the contribution of additional depression-linked factors that could affect TUT frequency, such as task difficulty or cue processing, the relationship between dysphoria level and task performance (errors of commission) and cue recognition memory were assessed.

### Hypotheses

The study tested the hypothesis that higher levels of dysphoria would be associated with greater tendency to spontaneously engage in negative relative to positive mental imagery-based future thinking. Spontaneous cognition is defined as TUTs generated without instructions to generate cognition of any particular kind.

The study further aimed to explore whether any observed relationship between dysphoria level and the tendency to generate emotional imagery future-TUTs was (a) specific to emotionally toned imagery future-TUTs (emotional specificity); (b) specific to imagery-based emotional future TUTs (representational format specificity); and (c) specific to future-oriented imagery TUTs (temporal orientation specificity). To verify (a) emotional specificity, the relationship between depression scores and neutral imagery future-TUT frequency will be assessed; to verify (b) representational format specificity, the relationship between depression scores and emotional future-oriented non-imagery TUTs will be assessed; and to verify (c) temporal orientation specificity, the relationship between depression score and emotional past-oriented imagery TUTs will be assessed. Given previous research on depression-linked reduction in positive relative to negative future imagery vividness when such imagery was deliberately generated, the present study also explored the vividness of spontaneously generated future imagery.

## Method

### Participants

Forty-five introductory psychology students at the University of Western Australia participated in the study. Recruitment was guided by a mass screening of more than 900 students using the Beck Depression Inventory (BDI-II; Beck, Steer, & Brown, 1996). Individuals scoring in the top, middle as well as bottom third of the BDI-II score distribution were invited to the study to obtain a wide spread of BDI-II scores in our sample. Sample size was guided by a priori power analysis using the R package “pwr” (Champely et al., 2018) based on (Cohen, 1988), for achieving 80% power to detect a medium effect size (guided by previous unpublished studies in the lab), *r* = 0.40 and *α* = 0.05. Participants received course credit for their participation in the study. The study was approved by the University of Western Australia’s Human Research Ethics Office (ethics code: RA/4/1/5243).

### Materials

#### Questionnaires

Depression symptom level was assessed using the Beck Depression Inventory-II (BDI-II; Beck, Steer, & Brown, 1996), a 21-item self-report scale measuring the level of depressed mood, cognition, and physical symptoms over the previous 2 weeks. Each item is rated on a 4-point scale, indicating increasing symptom severity. The BDI-II has high internal consistency (Storch, Roberti, & Roth, 2004) and concurrent validity, correlating highly with other measures of depressed mood, such as the DASS Depression subscale (Lovibond & Lovibond, 1995).

#### Task Unrelated Thinking task (TUTT) stimuli

Stimuli consisted of nine numerical digits and 270 singular words. The digits “1” to “9” were visually presented in Arial font (size 18). The words were selected from the Affective Norms for English Words corpus (ANEW; Bradley & Lang, 1999) based on normative ratings of emotional valence. Ninety words were selected from the top end of emotional valence scores (emotionally positive); ninety words were selected from the bottom end of emotional valence ratings (emotionally negative), and 90 were selected from mid-point of emotional valence ratings (emotionally neutral). As desired, words selected for the three word categories significantly differed in emotional valence scores, with positive words having higher scores than neutral words, and neutral words having higher scores than negative words, *F* (2, 240) = 1910.95, *p* < 0.001, *ƞ*^*2*^_*p*_ = 0.94. Negative and positive cue words did not differ in ANEW Arousal rating scores, *F* (1, 160) = 0.04, *p* = 0.85. All words were recorded by a female native-British English speaker and edited using Adobe Audition 3.0 (all sound files were 1000 ms in duration).

#### TUTT cue recognition memory test stimuli

The surprise cue recognition memory test administered following the TUTT was a pen-and-paper measure comprising 120 words, 60 of which were true cue words (20 negative, 20 positive, 20 neutral), and 60 were foil words (20 negative, 20 positive, 20 neutral). True and foil words did not differ in BNC word frequency, *F* = 1.793, *p* = 0.183. True and foil also did not differ in ANEW valence or arousal ratings (Bradley & Lang, 1999) across valence categories, all *F* (2, 114) < 2.034, all *p* > 0.135.

#### Experimental hardware

Task stimuli were presented via a 19-inch computer monitor set at 1280 × 1024 resolution (60 Hz refresh rate), with auditory stimuli presented via noise-cancelling headphones. A standard keyboard was used to record participants’ responses.

### Tasks

#### Task Unrelated Thinking task (TUTT)

The task unrelated thinking task (TUTT) was programmed using E-Prime 2.0 (Psychology Software Tools). The task was approximately 20–25 min in duration, and comprised 270 trials. On each trial, participants were presented with a 500 ms fixation cross, followed by the presentation of one numeral digit on the computer screen for 1000 ms, together with the simultaneous presentation of one word via headphones, lasting 1000 ms or less. Stimuli presentation was followed by a 1000 ms blank screen, making the inter-stimulus interval (ISI) 2500 ms, in line with previous literature (Smallwood, Fitzgerald, Miles, & Phillips, 2009). Number digits ranged from one to nine, appearing with equal probability in pseudo-random order (constrained to ensure no repeats of same digit on successive trials). Auditory cue words were presented in separate positive, neutral, and negative blocks, such that all negative words were presented together, and likewise for positive and neutral words, with presentation order of the three blocks counterbalanced across participants.

Participants were instructed to press the “3” key on the number keypad (labelled “Go”) as soon as a number other than 3 appeared on the screen (240 trials), and to withhold the button press when the numerical digit was the number 3 (30 trials). In addition, participants were also instructed to report task unrelated thoughts by pressing the “1” key on the number keypad (labelled with the drawing of a star sign). This key press paused the task to present rating questions. The first ratings asked participants to indicate (A) whether the task unrelated thought (TUT) was (1) mental imagery; (2) verbal linguistic; or (3) both; (B) how vivid the TUT was if it involved imagery; and/or how elaborate the TUT was if it was verbal-linguistic, on a 5-point Likert scale ranging from 1 (Not at all) to 5 (Extremely); (C) how emotionally negative or positive the thought content was on a 5-point scale ranging from 1 (Very negative) to 5 (Very positive); and (D) the temporal orientation of TUT content on an 8-point scale, consisting of 1 (past—years); 2 (past—months); 3 (past—days/weeks), 4 (present), 5 (future—days/weeks); 6 (future—months); 7 (future—years); 8 atemporal. The digit vigilance task resumed when participants had provided ratings for all questions.

#### TUTT cue recognition memory test

The test comprised a three-page paper-based task and participants were instructed to tick “yes” if the word was one the participant remembered hearing during the task, and “no” if they did not remember the word as one they heard during the task.

### Procedure

Participants were tested individually. Upon arriving at the laboratory, participants gave written informed consent and completed demographics information and the BDI-II questionnaire. Participants were then told that they were taking part in a concentration task, and were required to respond to a series of numerical digits on the screen while words played via the headphones. Participants were informed that on each trial, in addition to seeing numerical digits on the screen, they would also hear words from the headphones, which simply formed part of the task environment and that they were not required to respond to the words in this task.

Following instructions on how to complete the digit vigilance task, participants were informed that due to the monotonous nature of the task, most people’s minds tend to wander off frequently to think other things unrelated to doing this task right now. Participants were told that thinking about unrelated things was completely normal and they should not try to prevent it from occurring. Participants were then instructed to press the “star” key on the number pad whenever they realised they had been thinking about something unrelated to task. The experimenter then explained the differences between mental imagery and verbal-linguistic thoughts, and participants’ understanding were verified before completing 15 practice trials in the presence of the experimenter, where participants also practiced the TUT reporting component of the task even if they did not experience any TUTs during the short practice period.

The experimenter then left the room and participants completed the task, followed by the surprise

#### **Cue recognition memory** test

Finally, participants were debriefed and given course credit for their participation.

### Data analyses plan

#### Coding of TUTs

The frequency of future-oriented TUTs was extracted as a function of its emotional valence, representational format and temporal orientation, based on participants’ ratings. For representational format, TUTs reported to involve mental imagery or both mental imagery and verbal-linguistic thoughts were categorised as imagery TUTs, whereas TUTs involving only verbal-linguistic thought were considered non-imagery TUTs. For emotional valence, TUTs rated “4-positive” or “5-very positive” were categorised as positive, TUTs rated “1-very negative” or “2-negative” were categorised as negative, and TUTs rated “3-neutral” were categorised as neutral. For temporal orientation, TUTs rated 1 (past—years), 2 (past—months) or 3 (past—days/weeks) were categorised as past-TUTs; and all TUTs rated 5 (future—days/weeks), 6 (future—months) or 7 (future—years) were categorised as future-TUTs.

To investigate the representational format and temporal orientation specificity of any observed mood-congruent bias effects, TUTs are computed as a function of their emotional valence category (positive, negative, neutral), and as a function of their representational format (involving imagery, not involving imagery) and temporal orientation (past, present, future, atemporal). Given that the total number of TUTs is likely to differ across individuals, comparisons of TUT frequency as a function of TUT type within individuals (as well as across individuals) should take into account overall differences in TUT frequency. In addition, given the relatively low number of TUTs per category, computing percentages within categories (e.g. positive future TUTs involving imagery as a percentage of all future TUTs involving imagery) are likely to lead to large variations in percentages when the variation in the raw number is minimal, Therefore, to take into account general individual differences in TUT frequency and to minimize distortion in the data, all TUT frequencies are computed as percentages of the total TUTs for each participant.

For hypotheses testing, Spearman’s rank order^1^ correlational analyses were conducted between BDI-II scores and the following outcome variable bias scores. If the predicted relationship was found, planned post-hoc correlational analyses with positive and negative imagery future-TUT frequency scores were conducted separately to evaluate the presence of independent valence effects driving the results observed from bias scores.

In assessing dysphoria-linked bias in the tendency to generate emotional future TUTs involving mental imagery, to aid interpretation of valence bias scores, negative future imagery-TUTs are subtracted from positive future imagery-TUTs such that scores above zero indicate the presence of a positive bias, and scores below zero indicate the presence of a negative bias. The imagery future-TUT bias scores were computed via the following formula:

$${\text{Imagery Future-TUT Positivity Bias Score }}={\text{positive}}\;{\text{imagery future-TUTs }}\left( {\% {\text{ of total TUTs}}} \right){\text{ }}-{\text{negative}}\;{\text{imagery future-TUTs }}\left( {\% {\text{ of total TUTs}}} \right).$$

To assess the emotional specificity of observed depression-linked bias in imagery future-TUT tendency, emotionally neutral future-TUTs involving mental imagery will be correlated with BDI-II scores.

To assess the representation format specificity of observed depression-linked bias in imagery future-TUT tendency, non-imagery future-TUT positivity bias scores were computed via the following formula:

$${\text{Non-imagery Future-TUT Positivity Bias Score }}={\text{ positive non-imagery future-TUTs }}\left( {\% {\text{ of TUTs}}} \right){\text{ }} - {\text{ negative non-imagery future-TUTs }}\left( {\% {\text{ of TUTs}}} \right).$$

To assess the temporal orientation specificity of observed depression-linked bias in imagery future-TUT tendency, imagery past-TUT positivity bias scores were computed via the following formula:

$${\text{Imagery}}\;{\text{Past-TUT Positivity Bias Score }}={\text{ positive imagery}}\;{\text{past-TUTs}}\left( {\% {\text{ of total TUTs}}} \right){\text{ }}-{\text{ negative imagery}}\;{\text{past-TUTs}}\left( {\% {\text{ of total TUTs}}} \right).$$

To assess task performance, inattentional errors during the TUTT task as indicated by errors of commission (failure to withhold button press on trials where the number is “3”) was correlated with BDI-II scores. We also conducted additional exploratory analyses assessing the presence of group differences in cue processing, as measured by performance on a surprise recognition memory task (TUTT Cue Recognition Memory Test) at the end of the study (see Supplementary Materials A for analysis plan).

## Results

### Participant characteristics

Data from two participants were lost due to technical issues. Further, one participant did not report any TUTs and was thus excluded from data analysis. Of the remaining 42 participants, BDI-II scores ranged from 0 to 37 (*M* = 10.84; SD = 9.44).^2^ Average participant age was *M* = 21.55 years, SD = 6.08; average education level was *M* = 13.58 years, SD = 1.89. Of the 42 participants, 25 were female (59.5%), and 90% self-identified as native English speakers.

### Task performance

Performance on the SART is critically measured by the rate of errors of commission, which is the number of no-go trials on which a participant fails to withhold a keypress response. On average, commission error rate was *M* = 3.67, SD = 3.71. No significant relationship was found between BDI-II scores and errors of commission, *r*_s_ = 0.21, *p* = 0.18, indicating dysphoria level was not significantly related to the degree of attentional lapse in the task. Task performance can also be indexed using errors of omission, i.e. the number of go trials on which a participant fails to execute a keypress response. Omission error rate in the present study was 0, as all participants executed the required keypress on all *go* trials, either during the stimulus presentation window or in the subsequent mask window, similar to previous findings (Murphy et al., 2013).

Signal detection performance on the TUTT Cue Recognition Memory Test indicated no significant group differences in cue processing (see Supplementary Materials A for results).

### Spontaneous task unrelated thought frequency

#### General characteristics

Overall, participants generated a total of 763 task unrelated thoughts (TUTs), with an average of *M* = 18.17, SD = 11.20. BDI-II score was not related to total TUT raw frequency, *r*_s_ = 0.14 *p* = 0.38. With respect to emotional valence, a total of 162 TUTs (21.23%) were negative; 279 (36.57%) were positive; and 322 (42.20%) were neutral. With respect to representational format, a total of 488 TUTs (63.96%) involved mental imagery [mental imagery only: 269 (35.26%); mental imagery + verbal linguistic: 219 (28.70%), and 275 TUTs (36.04%) did not involve mental imagery (verbal-linguistic only)]. With respect to temporal orientation, a total of 242 TUTs (31.72%) were past-oriented; 224 (29.36%) were present-oriented; 147 (19.27%) were future-oriented; and 150 (19.66%) were atemporal in nature. Mean (raw and proportional to total) TUT frequency as a function of emotional valence, representational format and temporal orientation are presented in Table 1.

**Table 1:** Mean task unrelated thoughts (TUT) reported as a function of TUT Representation Format, TUT Temporal Orientation, and TUT Valence, presented as raw frequency and as percentages of total TUT frequency

TUT valence	TUT representation format											
	Involving mental imagery						Not involving mental imagery					
	Positive		Negative		Neutral		Positive		Negative		Neutral	
TUT temporal orientation	Raw (SD)	% (SD)	Raw (SD)	% (SD)	Raw (SD)	% (SD)	Raw (SD)	% (SD)	Raw (SD)	% (SD)	Raw (SD)	% (SD)
Atemporal	0.71 (1.63)	3.24 (6.29)	0.33 (0.57)	2.11 (4.32)	0.90 (1.21)	4.94 (6.69)	0.26 (0.50)	1.31 (2.76)	0.29 (0.64)	2.16 (6.00)	1.07 (1.42)	5.39 (6.73)
Past	1.91 (2.47)	10.40 (12.36)	1.00 (1.34)	4.97 (7.59)	1.81 (2.30)	8.59 (9.75)	0.33 (0.72)	1.53 (3.69)	0.33 (0.57)	2.26 (4.94)	0.38 (0.70)	2.28 (4.71)
Present	1.02 (1.30)	5.37 (6.44)	0.45 (0.89)	2.23 (4.38)	1.12 (1.71)	6.04 (8.14)	0.31 (0.60)	2.24 (4.66)	0.833 (1.36)	3.75 (5.81)	1.60 (2.35)	8.47 (11.53)
Future	1.57 (2.56)	9.96 (15.45)	0.43 (0.83)	2.10 (4.53)	0.36 (0.48)	3.06 (6.33)	0.52 (0.83)	4.02 (8.42)	0.19 (0.45)	0.70 (1.81)	0.43 (0.70)	2.89 (5.42)

#### Influence of auditory verbal cues

The valence of TUTs was influenced by the emotional tone of auditory cues, as emotionally negative TUTs were most frequently reported in the negative cue block; emotionally neutral TUTs were most frequently reported in the neutral cue block; and emotionally positive TUTs were most frequently reported in the positive cue block. See Supplementary Materials for additional data and explanation.

### Depression-linked bias in the tendency to experience emotional future-oriented TUTs involving mental imagery

**Fig. 1:**
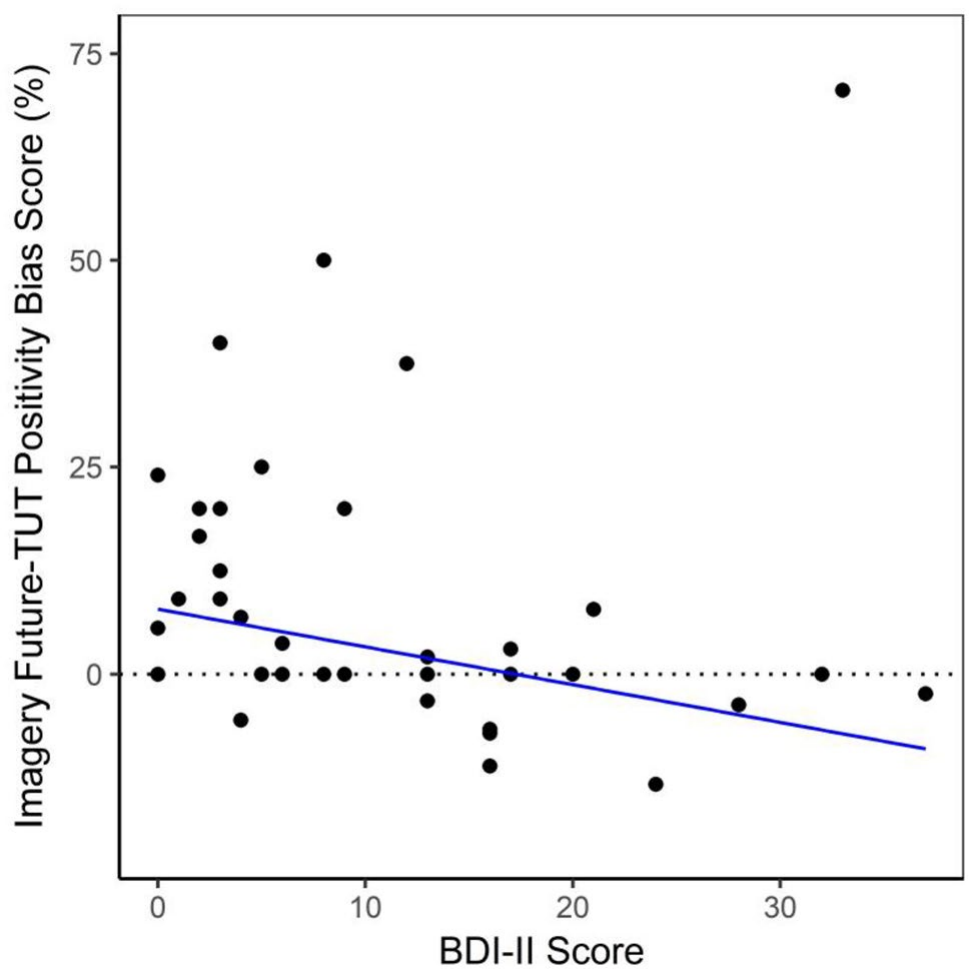
Scatterplot depicting a negative relationship between BDI-II scores and Imagery Future-TUT Positivity Bias Scores. A higher score indicates more positive imagery future-TUTs were generated than negative imagery future-TUTs (as proportional to total to TUT frequency). The trend line was fitted using robust regression using maximum likelihood estimates (instead of ordinary least squares estimates) to minimize the influence of outliers

Imagery Future-TUT Positivity Bis Score was correlated with BDI-II scores. As predicted, a significant negative relationship was found between BDI-II scores and Imagery Future-TUT Positivity Bias Scores, *r*_s_ (40) = − 0.42, *p* = 0.006. This relationship is depicted in Fig. 1. Planned post-hoc analysis revealed a significant positive relationship with the frequency of negative imagery-based future TUTs, *r*_s_ (40) = 0.46, *p* = 0.002; and a smaller negative relationship between BDI-II scores and the frequency of positive imagery-based future TUTs, *r*_s_ (40) = − 0.28, *p* = 0.07. This indicates that higher dysphoria levels are associated with greater tendency to generate negative imagery-based future-TUTs, and to a smaller extent, reduced tendency to generate positive imagery-based future-TUTs.

#### Testing the emotional specificity of observed effects

To assess whether the observed negative relationship between BDI-II scores and Imagery Future TUT Positivity Bias Scores is specific to emotional TUTs, BDI-II scores were correlated with the percentage of Imagery Future TUTs that were emotionally neutral. No significant relationship between BDI-II scores and neutral imagery future TUT frequency was found, *r*_s_ (40) = 0.05, *p* = 0.75, indicating the dysphoria-linked bias does not appear to reflect biases in the generalized tendency to experience future-TUTs involving mental imagery.

#### Testing the representational format specificity of observed effects

To assess whether the observed depression-linked bias is specific to future-oriented emotional TUTs involving mental imagery, the relationship between depression symptom level and the frequency of future-oriented emotional TUTs not involving mental imagery was assessed. No significant relationship was found between BDI-II scores and Non-imagery Future-TUT Positivity Bias Scores, *r*_s_ (40) = − 0.10, *p* = 0.53. This indicates that the dysphoria-linked decrease in positive bias for future-TUTs was specific to future-TUTs involving mental imagery.

#### Testing the temporal specificity of observed effects

The relationship between depression symptom level and the frequency of past-oriented emotional TUTs involving mental imagery was examined to assess whether the observed depression-linked valence bias was specific to future-oriented emotional TUTs involving mental imagery. BDI-II score was found to be not significantly related to Imagery Past-TUT Positivity Bias Scores, *r*_s_ (40) = − 0.02, *p* = 0.89. The results indicate dysphoria level was not associated with a mood-congruent bias in the frequency of emotional past-oriented TUTs. The observed depression-linked reduction in positive relative to negative future-oriented TUTs involving mental imagery was therefore specific to TUTs with a future temporal orientation

#### Depression-linked bias in emotional imagery future-TUT vividness

No significant relationship was found between BDI-II scores and mean vividness ratings for negative future imagery-TUTs, *r* (40) = 0.09, *p* = 0.80; or mean vividness ratings for positive future imagery-TUTs, *r* (40) = − 0.09, *p* = 0.67, indicating dysphoria level is not related to the vividness of emotional future imagery-TUTs.

## Discussion

The present study investigated the presence of depression-linked bias in the tendency to engage in spontaneous mental imagery-based future thinking. As hypothesized, results showed that when not instructed to deliberately generate task unrelated cognition of any kind, higher depression scores were associated with lower positive bias in the frequency of imagery-based emotional future-oriented task unrelated thoughts (TUTs). This depression-linked reduction in positive future imagery bias was driven by an increasing tendency to generate negative future imagery, and a decreasing tendency to generate positive future imagery. While the positive relationship between BDI-II score and the proportional frequency of positive future imagery approached statistical significance, the magnitude of the relationship (*r*_s_ = − 0.28) can be considered of moderate effect size in individual difference research (Gignac & Szodorai, 2016). Importantly, no relationship was observed between depression scores and the frequency of emotionally neutral future imagery, indicating the observed depression-linked effect was specific to emotional future imagery. In addition, depression scores were not related to future-oriented TUTs that did not involve mental imagery (verbal-linguistic representation only), indicating that the observed depression-linked bias was specific to imagery-based future cognition. Finally, depression scores were not related to the frequency of imagery-based past-oriented TUTs, indicating that the observed bias was specific to future imagery.

## Implications

Results from the study indicate that when engaging in off-task mindwandering, individuals with higher levels of symptoms of depression may be more likely to experience negative relative to positive mental imagery representations of the future than those with lower levels of depression symptoms. What might this mean in daily life? Previous experience sampling research suggests that up to half of waking mental life may consist of thoughts unrelated to the task at hand that occur spontaneously, without explicit purpose or intention (Killingsworth & Gilbert, 2010; McVay, Kane, & Kwapil, 2009). The same pattern has been found in a diary study examining naturally occurring mental imagery-based cognition (Kosslyn, Seger, Pani, & Hillger, 1990). As such, given the capacity for mental imagery to evoke emotional response (Ji et al., 2016), a greater tendency to experience task unrelated negative relative to positive future imagery is likely to contribute to elevated negative emotional states and reduced positive emotional states, thereby contributing to emotional disturbance in depression.

The present research extends previous literature on future thinking in depression by providing empirical evidence that the depression-linked biases observed when participants were instructed to deliberately think of emotional future events (Holmes et al., 2016; MacLeod, 2016), which are also observed when participants are not instructed to deliberately generate task unrelated cognition of any kind. The present findings are consistent with theoretical accounts that implicate maladaptive spontaneous cognition in depression vulnerability, in part due to the potential of such thoughts to promote feelings of hopelessness and pessimistic beliefs about the future (Marchetti, Koster, Klinger, & Alloy, 2016; Miloyan, Pachana, & Suddendorf, 2014). Importantly, results from the present study suggest that it may be specifically the tendency to generate imagery-based mental representations of the future that is specifically implicated in depression. The findings also corroborate previous retrospective self-report studies that indicate impoverished experiences of positive relative to negative future imagery in depression (Weßlau & Steil, 2014) and dysphoria (Weßlau et al., 2015) in daily life. If future studies using clinical samples replicate the present findings, this would indicate that depression is associated with a reduction in the general likelihood of experiencing positive relative to negative mental representations of the future involving mental imagery.

Furthermore, the present study also extends research on spontaneous past-oriented cognition by examining the role of mental representational format. It is somewhat surprising that the present study did not find any relationship between spontaneous past-oriented mental imagery and dysphoria level, given previous findings concerning past-oriented spontaneous cognition using similar sustained attention to response tasks. Such studies have found a greater tendency in past-oriented task unrelated thinking to be associated with depressed mood (Smallwood & O’Connor, 2011; Smallwood, O’Connor, Sudbery, & Obonsawin, 2007) and depression (Hoffmann et al., 2016). However, it is worth noting that such studies did not employ the use of cues to trigger TUTs. Research comparing SART tasks with concurrent verbal cues, relative to the same task without concurrent verbal cues, found that verbal cues increased the frequency of past-oriented TUTs and decreased the frequency of future-oriented TUTs, but did not modulate the frequency of present-oriented or atemporal TUTs (Vannucci, Pelagatti, & Marchetti, 2017). Previous research employing verbal cues has found that past-oriented TUTs occurred more frequently than future-oriented TUTs overall, but the tendency to spontaneously experience past-oriented TUTs was not modulated by dysphoria (Plimpton et al., 2015). Although Plimpton et al. (2015) did not delineate the nature of past-oriented TUTs based on their representational format, consistent with their findings, post-hoc exploratory analysis of the present data indicated no significant relationship between BDI-II scores and the frequency of past-oriented TUTs overall (*r*_s_ = − 0.19, *p* = 0.21).

It is also worth noting that the present study found no relationship between dysphoria level and the frequency of negative or positive TUTs not involving mental imagery (all *r*_s_ < |0.11|, all *p* > 0.63). This may at first appear surprising given that depression is associated with rumination, a predominantly verbal-linguistic form of internally generated passive thinking mode focused on the causes and negative consequences of one’s problems and depressive symptoms (Nolen-Hoeksema, 1991). However, more recent research indicate that sensory-perceptual representations are also highly prevalent in depressive cognition (Moritz et al., 2014), with more recent measures of repetitive negative thinking (including rumination) assessing the frequency of both thoughts (verbal-linguistic) and images (McEvoy, Hayes, Hasking, & Rees, 2017; Watkins, Moulds, & Mackintosh, 2005). As such, it is possible that depression-linked biases in past-oriented spontaneous cognition are more readily observable at clinical levels of depression, but such thoughts are likely to involve both imagery and verbal-linguistic representations. Of course, given that TUTs that did not involve any mental imagery comprised only 36% of total TUTs, it is possible that floor effects in the frequency of such TUTs make possible relationships difficult to detect. For example, it is possible that the exclusive use of auditory cues preferentially promoted the natural occurrence of mental imagery-based cognition, relative to purely verbal-linguistic cognition due to modality-specific task load that disproportionately taxes the phonological loop component of working memory (Repovs & Baddeley, 2006). Future studies could test this possibility by comparing two versions of the present task, one presenting auditory cues, and one presenting visual cues.

## Limitations

A possible limitation of the task is that self-reporting of task unrelated thinking may have influenced the natural occurrence of such thinking compared to probe-caught approaches. Both self- and probe-caught approaches prime participants’ awareness of off-task thinking, and while self-caught mindwandering relies on participants’ monitoring of their own conscious experience (Giambra, 1993; Schooler, 2002), previous research suggests that it does not alter phenomenological experience during undemanding cognitive tasks (Schooler, 2002; Smallwood, Baracaia, Lowe, & Obonsawin, 2003). However, previous research employing both self-caught as well as probe-caught mindwandering found thought probes caught additional TUTs not self-reported by participants (Schooler, 2002), indicating that studies using only self-caught TUT reporting may still underestimate the true occurrence of TUTs. In addition, even with the employment of cues and a “self-caught” approach to assessing TUT occurrence, frequencies of future-oriented TUTs were still low when analysed as a function of emotional valence and representational format. Future studies investigating individual differences in spontaneous cognition frequency may benefit from employing both self-caught and probe-caught approaches, and/or to seek to specifically evoke higher future focus during the task, such as priming current concerns.

In addition, the present results should be interpreted with caution as the study had a relatively small sample size, particularly due to the loss of three participants for the main analysis (with even fewer participants in some analyses due to the lack of TUTs reported that are of the category under examination), and four participants for the cue recognition memory task, leading to reduced power to detect true effects. Further validation of the present results using larger samples will be required to assess the reliability of the present results.

Further, it is likely that both the self-caught and probe-caught approaches to assess task unrelated thinking impacts on the natural flow of task unrelated thinking by requiring participants to report on the occurrence of task unrelated thinking. The present results therefore must be interpreted within the context of a controlled laboratory environment designed to encourage the occurrence of off-task thinking as well as reporting of its occurrence. Further, it must be acknowledged that asking participants to answer specific questions about the nature of task unrelated thinking (e.g. representational format, valence, and temporal orientation) may influence the general occurrence of task unrelated thinking. However, given that no emphasis was drawn to specific types of TUTs within each characteristic category (i.e. thoughts of a particular representational format, valence or temporal orientation), observed biases in the occurrence of TUTs within such characteristic categories are unlikely to be influenced by the need to report on the presence or nature of task unrelated thinking in general.

Finally, another limitation of the study is that the *spontaneous* occurrence of TUTs was operationalised as those occurring without instructions to deliberately generate task unrelated cognition of any kind, but the extent to which such TUTs occurred with or without intention was not further assessed. Given recent research on the dissociation between unintentional and intentional mindwandering (Seli, Carriere, & Smilek, 2015; Seli, Risko, & Smilek, 2016), delineating between depression-linked differences in the tendency to intentionally versus unintentionally engage in emotional future thinking may provide further insight into nature of cognitive biases underlying depression.

### Future directions

To further illuminate the role of biased tendency to spontaneously engage in emotional imagery-based future thinking, future research should replicate the present effects in clinical populations. In addition, while the present study used emotional words that were not selected to be of specific relevance to depression, previous research indicates that self-relevance of cues is associated with greater mood-congruent bias effects in depression (cf. Wisco, 2009). Therefore, future studies could test whether the use of depression-relevant emotional cues are associated with stronger outcomes, particularly in clinical populations. In addition, to advance the present line of research further, future research could investigate the degree to which the occurrence of spontaneous emotional imagery-based future thinking contributes to on-going emotional experience in depression, such as assessing state emotion change before, during, and after the TUTT task.

The present study showcases a novel laboratory paradigm for eliciting TUTs that can assess depression-linked individual differences in the tendency to experience future-oriented imagery. Such tasks can fruitfully be used within translational studies, such as emerging interventions that aim to tackle depression via training cognition to be more positive and less negative, such as future cognitive bias modification of interpretation (CBM-I) research (for a review, see Menne-Lothmann et al. 2014). Future CBM-I training studies could employ the present laboratory task as a measure of far transfer to assess the impact of interpretation bias training effects on the spontaneous tendency to experience positive relative to negative thoughts.

It would be important for future research to investigate the underlying causes of depression-linked biases in the tendency to imagine emotional experiences in the future, such as the role of negative interpretations of ambiguous information and negative expectations of the future. At the same time, future research should also examine the downstream impacts of biased tendency to engage in emotional imagery-based future thinking in dysphoria and depression. Using the same experimental paradigm, future studies could measure the degree to which variation in state emotions after the task are accounted for by the occurrence of negative and positive future imagery during the task. Further, given the growing theoretical focus and empirical evidence that imagery-based mental simulations of future events facilitate decision-making, planning and self-regulatory behaviour (Bulley, Henry, & Suddendorf, 2016; Gilbert & Wilson, 2007; Kappes & Morewedge, 2016; Seligman, Railton, Baumeister, & Sripada, 2013; Szpunar, Spreng, & Schacter, 2014; Taylor, Pham, Rivkin, & Armor, 1998), future research should examine how biases in imagery-based future thinking impact decision-making, planning and self-regulation in depression.

## Electronic supplementary material

Below is the link to the electronic supplementary material.

Supplementary material 1 (DOCX 458 KB)

## Footnotes

1 Non-parametric statistical tests of correlation were used as the data were heavily skewed to the right, such that no corrections (e.g. log transformation) could normalize the distribution, rendering it unamenable to parametric correlational analyses.

2 1 Based on cutoffs used in previous research using the BDI-II to assess high and low levels of dysphoria (e.g. Berna, Lang, Goodwin, & Holmes, 2011; Holmes, Lang, et al., 2008), our participant sample had approximately equal numbers with low dysphoria (BDI-II < 6, *n* = 15), high dysphoria (BDI-II > 13, *n* = 12), with the rest falling in between low and high dysphoria (*n* = 15).
