# Supplementary material for: Spontaneous cognition in dysphoria: reduced positive bias in imagining the future
Source: Psychol Res. 2018 Aug 10;83(4):817–31. doi: 10.1007/s00426-018-1071-y (PMC6529377; doi:10.1007/s00426-018-1071-y)
Supplement: Supplementary file 1 — Supplementary material 1 (DOCX 458 KB) [file 426_2018_1071_MOESM1_ESM.docx]

**Supplementary Materials**

**A) TUT Task Cue Recognition Memory Test.**

**Analysis plan:**

To assess whether the groups differed in their processing of cues they were exposed to during the TUTT task, overall and as a function of cue valence, recognition memory task performance was analysed using signal detection analysis (Swets, Tanner Jr., & Birdsall, 1961). Performance was assessed via computation of d-prime (d’) scores using the formula d’= ZHit– ZFA, where “Hit” (hit rate) is the number of correctly recognised cue words as a proportion of the total number of hits and misses (incorrect rejection of cue words), and “FA” (false alarm rate) is the number of false alarms (foils misrecognised as a true word) expressed as a proportion of the total number of false alarms plus corrected rejected foils. Spearman correlational analysis will be conducted between BDI-II score and the relative proportion of hits minus false alarms (d’) for negative, neutral and positive cue words.

**Results:**

Four participants did not complete the cue memory data correctly. For the remaining 38 participants, no significant relationships were found between BDI-II scores and d’ scores in the Negative Cue block, *r* _s_ = - 0.16, *p* = 0.34, or Positive Cue Block, *r* _s_ = - 0.08, *p* = 0.64, indicating dysphoria level was not significantly related to the probability that cues from negative versus positive blocks were better remembered.

**B) Congruence between cue valence and mental imagery valence**

The task used by the present study research does not ask participants to indicate whether their task unrelated thoughts (TUTs) were related to the auditory cues they heard during the task. However, cues were presented in valence-congruent blocks, therefore the influence of cues can be assessed by checking the valence congruence between cues and TUTs reported. Figure 2 below shows the total number of emotionally negative, neutral and positive TUTs reported in each block (negative cue block, neutral cue block, positive cue block). As can be seen, emotionally negative TUTS were most frequently reported in the negative cue block; emotionally neutral TUTs were most frequently reported in the neutral cue block; and emotionally positive TUTS were most frequently reported in the positive cue block. This congruence between cue valence and TUT valence indicates that TUTs were influenced by the emotional tone of cues.


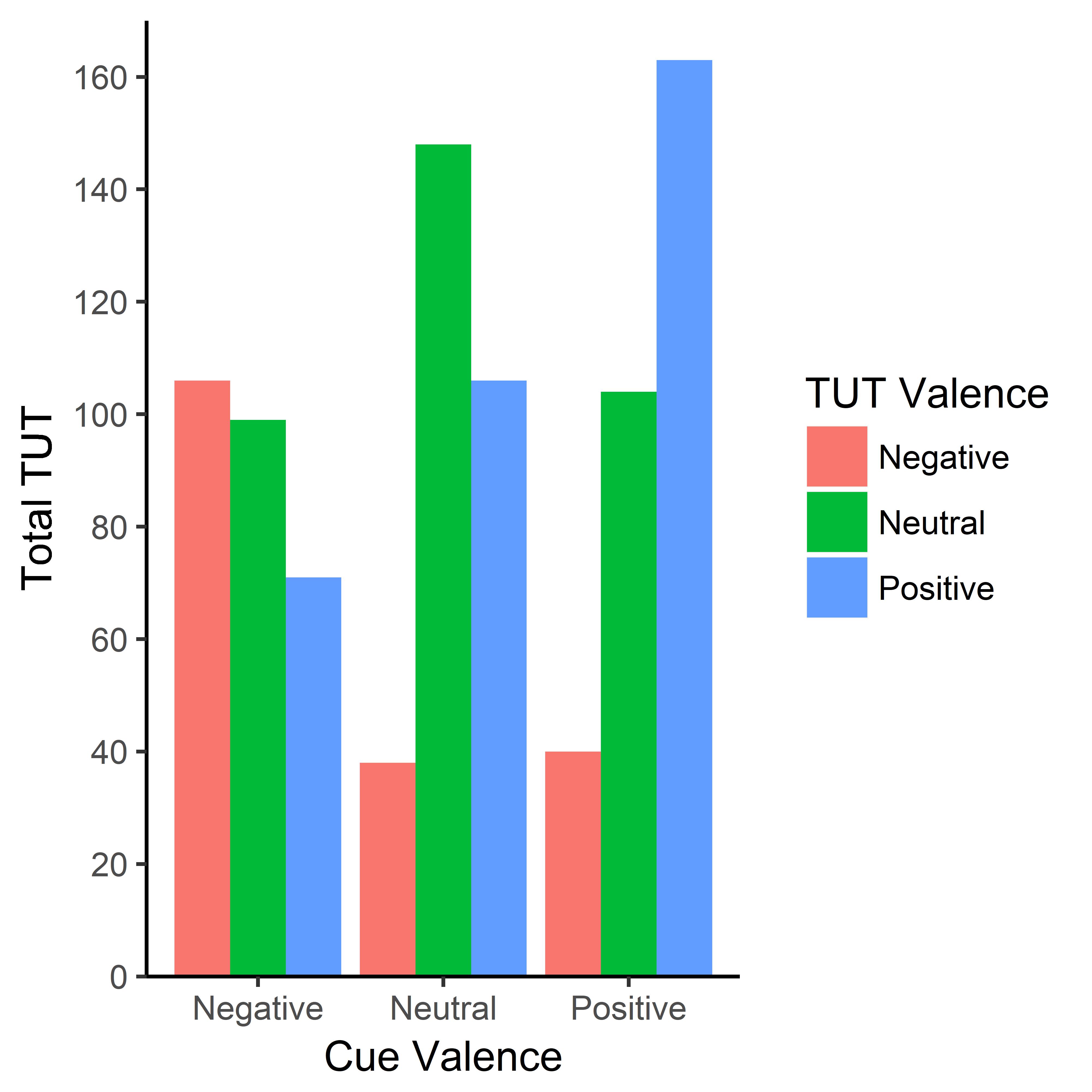


*Figure 2*: Bar graph depicting the total number of emotionally negative, neutral and positive TUTs reported in each block (negative cue block, neutral cue block, positive cue block).
